# Letter to the editor: Not inferior but rather superior long-term disease-free survival after laparoscopic versus open total mesorectal excision for low and mid rectal cancer!

**DOI:** 10.1007/s00384-021-04087-2

**Published:** 2022-01-06

**Authors:** Andreas D. Rink

**Affiliations:** Division of Minimally Invasive Oncologic Surgery, Department of General, Visceral and Transplant Surgery, Essen University Hospital, Hufelandstr. 55, 45147 Essen, Germany

Dear Editor:

With great interest I read the article “Mid-and low-rectal cancer: laparoscopic versus open treatment – short and long-term results. Meta-analysis of randomized controlled trials” recently published in the November issue of the *International Journal of Colorectal Disease* by Schietroma and co-workers [1]. I appreciate the authors’ idea of evaluating the role of laparoscopic surgery specifically for the treatment of mid and low rectal cancer. The authors state to have performed a meta-analysis of randomized controlled trials comparing laparoscopic versus open surgery for extraperitoneal rectal cancer. They convincingly present several advantages of the laparoscopic approach in terms of non-oncologic short-term outcomes. Furthermore, they demonstrated equivalence of laparoscopic surgery for most oncologic outcome criteria. However, the oncologic long-term outcome in terms of disease-free survival (DFS) is claimed to be inferior after laparoscopic surgery.

This latter statement is presented as a result of a meta-analysis of four trials that have investigated long-term DFS, as presented in table 11 of the article [2–5]. Looking to these studies in detail, one recognizes first that the study presented by Baik in 2011 [3] was a case-controlled rather than a randomized trial. According to the authors’ inclusion criteria, it should have been completely excluded from the analysis. Anyhow, the presented 5-year DFS rates for laparoscopic surgery were more than 5% better than for open surgery (80.8% laparoscopic versus 75.6% open). In the ACOSOG Z6051 trial [5], the DFS at 4 years was 75.2% (95% CI 69.6–81.1%) versus 73.2% (95% CI 67.2–79.8%) in the laparoscopic and open groups, respectively, which was quite similar but indicates superiority rather than inferiority of laparoscopic surgery. The same is true for the study presented by Lujan in 2009 [2]: DFS at 5 years was 84.8% (95% CI 75.4–94.2%) after laparoscopic versus 81.0% (95% CI 71.4–90.6%) after open surgery, respectively. Again, this difference was not significant but rather indicating superiority than inferiority of laparoscopic surgery. Finally, Ng and co-workers presented a 5-year DFS of 83.3% for patients operated on by laparoscopy and only 74.5% for those operated on with the open technique. This difference of almost 9% in benefit of laparoscopy was also not significant (*p* = 0.114), but it implies again a benefit of the laparoscopic approach, as impressively demonstrated in the Kaplan-Meyer survival curves presented in the original article published by Ng and colleagues [4].

Thus, not even one of the four trials processed in the meta-analysis indicates a trend towards inferiority of the laparoscopic approach but all rather indicate equivalence if not superiority. Accordingly, the pooled data of the studies as presented in table 11 of the authors’ manuscript demonstrate 79% (409/518) long-term DFS for laparoscopy and only 74% (410/553) for open surgery. It is hard to understand how the authors could come to the result that “4 and 5 years disease-free survival were statistically higher after open surgery.”

It is a pity that this wrong statement is presented as a key message of a peer-reviewed article in a well-known international journal. The statement is likely to be cited in the future frequently as evidence for oncologic inferiority of the laparoscopic TME, although it obviously results from an error in the authors’ analysis.
